# Application of a qPCR Assay in the Investigation of Susceptibility to Malaria Infection of the M and S Molecular Forms of *An. gambiae* s.s. in Cameroon

**DOI:** 10.1371/journal.pone.0054820

**Published:** 2013-01-22

**Authors:** Anne Boissière, Geoffrey Gimonneau, Majoline T. Tchioffo, Luc Abate, Albert Bayibeki, Parfait H. Awono-Ambéné, Sandrine E. Nsango, Isabelle Morlais

**Affiliations:** 1 Unité mixte de recherche MIVEGEC (IRD 224- CNRS 5290-UM1-UM2), Institut de Recherche pour le Développement, Montpellier, France; 2 Laboratoire de Recherche sur le Paludisme, Organisation de Coordination pour la lutte contre les Endémies en Afrique Centrale, Yaoundé, Cameroon; Université Pierre et Marie Curie, France

## Abstract

*Plasmodium falciparum* is the causative agent of malaria, a disease that kills almost one million persons each year, mainly in sub-Saharan Africa. *P. falciparum* is transmitted to the human host by the bite of an *Anopheles* female mosquito, and *Anopheles gambiae sensus stricto* is the most tremendous malaria vector in Africa, widespread throughout the afro-tropical belt. *An. gambiae* s.s. is subdivided into two distinct molecular forms, namely M and S forms. The two molecular forms are morphologically identical but they are distinct genetically, and differ by their distribution and their ecological preferences. The epidemiological importance of the two molecular forms in malaria transmission has been poorly investigated so far and gave distinct results in different areas. We have developed a real-time quantitative PCR (qPCR) assay, and used it to detect *P. falciparum* at the oocyst stage in wild *An. gambiae* s.s. mosquitoes experimentally infected with natural isolates of parasites. Mosquitoes were collected at immature stages in sympatric and allopatric breeding sites and further infected at the adult stage. We next measured the infection prevalence and intensity in female mosquitoes using the qPCR assay and correlated the infection success with the mosquito molecular forms. Our results revealed different prevalence of infection between the M and S molecular forms of *An. gambiae* s.s. in Cameroon, for both sympatric and allopatric populations of mosquitoes. However, no difference in the infection intensity was observed. Thus, the distribution of the molecular forms of *An. gambiae* s.s. may impact on the malaria epidemiology, and it will be important to monitor the efficiency of malaria control interventions on the two M and S forms.

## Introduction

Malaria remains the most important vector-borne disease in sub-Saharan Africa, affecting each year over 200 million people and killing almost one million deaths, mostly children under five and pregnant women [1]. The disease is caused by the protozoan parasite *Plasmodium falciparum,* and the parasite is transmitted by the bite of a female *Anopheles* mosquito. Malaria control is a priority in the Millenium Development Goals (MDGs) [2], and substantial funds from the Global Fund and the President’s malaria initiative (PMI) allowed implementation of integrated activities for effective antimalarial interventions. Malaria control efforts focus on combined interventions, among them large coverage of insecticide-treated mosquito nets (ITNs) and treatment with effective antimalarial drugs. Over the past decade, significant progress has been achieved in reducing the burden of malaria in many endemic countries [3]. Unfortunately, the wide use of ITNs has lead to an increase of insecticide resistance in mosquito populations, and in Asia, resistance to artemisinin has already been reported [4], [5], [6], hampering the promising results in the fight against malaria.

*An. gambiae* s.s. is the most efficient malaria vector in sub-Saharan Africa. The mosquito has a marked human feeding preference, a high susceptibility to *Plasmodium*, and is present at high densities during malaria transmission seasons [7], [8], [9]. *An. gambiae* s.s. was subdivided into two distinct molecular forms, namely M and S forms, based on polymorphisms in the ribosomal DNA [10]. Reproductive isolation and genetic divergence between the two molecular forms support that M and S are cryptic species [11], [12], [13], [14], [15], [16], [17], [18]. In the wild, the M and S forms colonize different ecological niches, the S form being more adapted to arid environments, but the two forms can also be found in sympatry [19], [20]. The relative susceptibility of the M and S forms to malaria infection has been poorly investigated and gave rise to different results, in Senegal the S form was more susceptible than the M one, but no difference between the two forms was observed in Mali [21], [22]. Further studies are needed at larger scale to identify genetic and/or ecological factors that determine transmission by natural vector populations; this has important implications to target vector control.

*P. falciparum* parasites have to go through a series of developmental steps during their life cycle within the mosquito vector [23], and the passage of the parasites through the midgut epithelium represents a critical step where important bottleneck occurs. Malaria parasites have to cross the midgut epithelium, where ookinetes transform into oocysts, and there, they encounter severe losses due to the mosquito immune responses, the midgut microbiota and other factors [24], [25], [26]. Thus, the mosquito midgut is an attractive site for novel targeted malaria control strategies, such as transmission blocking vaccines or drugs (TBVs, TBDs).

Current methods to study *P. falciparum* transmission in the mosquito vector are based on parasite detection upon the dissection of mosquitoes and the microscopic observation of midguts. In experimental settings, *P. falciparum* midguts are examined 6 to 8 days upon the infection, when oocysts are big enough to be detected at magnification ×200, and the mosquito infection is measured by the count of oocysts developed in the midgut. In the midgut, the parameters of infection are based on infection prevalence and intensity, where prevalence of infection (IP) is defined as the proportion of mosquitoes harboring at least one oocyst, and infection intensity (II) as the number of oocysts per mosquito among mosquitoes with ≥1 oocyst. At this stage, the infection prevalence is the key parameter for measuring malaria transmission, because a single oocyst is sufficient for the mosquito to become infectious and transmit the disease.

Determining the mosquito infection parameters is labor intensive and time-consuming; every single mosquito needs to be dissected alive and processed freshly. In addition, microscopic examination has limitations; midguts with only one oocyst can be mis-scored. New tools for efficient high-throughput screening of infection in mosquitoes are then needed. Molecular tools have been largely developed for the diagnosis of *Plasmodium* in clinical samples and for research purposes, allowing the detection of *Plasmodium* in cases with low parasitaemias, as well as mixed infections of malaria [27], [28], [29], [30], [31], [32], [33], [34]. PCR-based methods were subsequently applied for the detection of *Plasmodium* in mosquitoes [35], [36], [37], [38], increasing the sensitivity of the routine microscopy. Most of these methods are targeting the small subunit (SSU) rRNA gene, which has 4–8 copies per individual parasite [39]. However, these diagnostic tools more often require multiple reactions or expensive fluorescent markers that are not affordable for national control programs in malaria endemic areas.

In this study, we have developed a quantitative PCR assay to detect *P. falciparum* in mosquitoes infected with natural isolates of parasites. The real-time assay is based on the amplification of a fragment of the subunit 1 of mitochondrial cytochrome c oxidase (cox1) gene, and allows specific amplification in a single round reaction. We targeted the cox1 gene to increase the sensitivity of the assay, indeed *P. falciparum* parasites contain numerous mitochondrial genomes, with numbers of mitochondria differing between sexual and asexual stages [40], [41]. We evaluated the validity of this assay to measure parameters of infection in the mosquito midgut by comparison with conventional PCR. The cox1 qPCR assay was then used to screen *P. falciparum* infection in wild mosquitoes experimentally infected on blood from gametocyte donors, highlighting differences in the susceptibility to malaria infection among the M and S forms of *An. gambiae* s.s. in Cameroon.

## Materials and Methods

### Ethics Statement

All procedures involving human subjects used in this study were approved by the Cameroonian national ethical committee (statement 099/CNE/SE/09). Children identified as gametocyte carriers were enrolled as volunteers after their parents or legal representative had signed a consent form. All necessary permits were obtained for the described field studies (statement 099/CNE/SE/09). Collections did not involve any protected species, and the collecting sites were either public areas or private gardens; in this later case, the owner gave the permission for collection.

### Mosquito Collections

*An. gambiae* mosquitoes were collected at L4 and pupae stages in larval habitats from four localities near Yaoundé (Cameroon), using the dipping method [42]. Water collections were placed in 5-liters containers and brought back to the insectary at Organisation de Coordination pour la lutte contre les Endémies en Afrique Centrale (OCEAC, Yaoundé, Cameroon). Immature stages were inspected visually, and non anopheline larvae and eventual predators removed. Larvae from each sampled locality were placed in a 3-liters plastic bucket in water from their breeding site and reared for 2 days. Pupae were picked daily using a 5 ml plastic pipetor and kept in a 20 ml plastic glass inside a 30×30 cm cage for emergence. Adult mosquitoes were maintained in standard insectary conditions and provided with a 6% sterile sucrose solution.

### Experimental Infections

Experimental infections were performed as previously described [43], [44]. The procedure includes replacement of the volunteer serum by a non-immune AB serum to avoid human transmission blocking factors. Female mosquitoes, 2 to 5 days old, starved for 24 h prior feeding, were allowed to feed for 35 minutes. Unfed and partially fed mosquitoes were removed by mouth aspiration and discarded. Fully engorged females mosquitoes were kept in insectary until dissections at 8 days post infection. Midguts were dissected in sterile phosphate-buffered saline solution under a binocular microscope and kept frozen individually.

### DNA Extraction, *P. falciparum* Detection, and Characterization of *An. gambiae* s.s. Mosquitoes

A synchronous *P. falciparum* (3D7 strain) culture maintained at a 5% hematocrit of human red blood cells (∼5×10^5^ rbc/µl) in RPMI medium and containing asexual ring-stage parasites (haploid genomes) with a 12% parasitaemia was kindly provided by Dr. Berry (Rangueil Hospital, Toulouse, France). The number of genomes per microliter of culture was estimated as 5×10^5^×0.12. DNA was extracted from 200 µl of the parasite culture and served to build for calibration curve. DNAs from mosquito midguts and cultured parasites were extracted using the DNeasy Blood & Tissue Kit (Qiagen, Valencia, CA), according to the manufacturer’s instructions, and resuspended in 20 µl and 200 µl volumes of sterile H_2_O, respectively. A conventional PCR for the identification of malaria infections in the midguts was run using a *P. falciparum*-specific PCR amplifying a 440 bp fragment of the cox1 gene, as described by Fabre *et al* [45]. Molecular forms of field-caught *An. gambiae* s.s. mosquitoes were determined using the PCR-RFLP protocol developed by Fanello *et al* [46].

### *P. falciparum* Detection Using a Quantitative PCR Assay Targeting the cox1 Gene

The quantitative PCR assay was performed using EvaGreen dye (Euromedex, Souffelweyersheim, France.) and samples were run on a 7300 Real-Time PCR System (Applied Biosystems, Foster City, CA, USA). The qPCR assay is targeting a 120 bp sequence of the cox1 gene, located inside the target sequence of the conventional PCR, forward primers of both assays overlapping. Forward and reverse primer sequences were qPCR-PfF 5′-TTACATCAGGAATGTTATTGC-3′ and qPCR-PfR 5′-ATATTGGATCTCCTGCAAAT-3′, respectively [47], [48]. The specificity of the primer set was checked by running qPCR reactions with DNAs (20 ng/µl) from *P. vivax*, *P. malariae, P. ovale* and *An. gambiae*. Reaction mixtures were prepared in a 10 µl final volume containing 1 µl of template DNA, 1× HOT Pol EvaGreen qPCR Mix Plus ROX, and 600 nM of each primer. The PCR conditions consisted of an initial melting cycle at 95°C for 15 minutes, followed by 40 cycles of amplification at 95°C for 15 s (denaturation) and 58°C for 30 s (annealing, extension). Dissociation curves were generated after the final amplification cycle by denaturating the amplicons at 95°C for 15 sec, cooling the temperature to 60°C for 30 sec, and then increasing the temperature up to 95°C at a ramp rate of 0.03°C/sec. Dissociation curves were used to estimate the specific melting temperature for each reaction. The specificity of the reaction was verified on a 2% (w/v) agarose gel stained with ethidium bromide.

### Standard Curve and Absolute Quantification

A standard curve was generated from 10-fold serial dilutions of the cultured parasite DNA corresponding to a range of 6 to 60 000 genome/µl. Serial dilutions served as templates for qPCR reactions and were added in duplicate in each 96-well reaction plate. A total of 53 amplification curves were used to obtain the standard curve.

The efficiency of amplification curves and absolute quantification of parasites in starting templates were determined using the LinRegPCR software [49], [50]. The software determines the baseline fluorescence for each reaction and applies a baseline correction. An optimal window-of-linearity (W-o-L) is defined from the data points in the log-linear phase of the amplification curve and a regression line determined from the W-o-L. The estimate of the starting concentration (N0) in each sample was directly computed from the intercept of the regression line [50]. The mean PCR efficiency for each sample is derived from the slope of the regression line. The means of amplification efficiencies were compared between the parasite culture and the mosquito samples to check for unequal amplifications due to putative presence of inhibitors or to competition with the mosquito DNA; means were compared using a Wilcoxon test. The starting concentration of the sample is expressed in arbitrary fluorescence units and was converted to the number of genomes/µl using a calibration curve built from the 10-fold serial dilution dataset.

### qPCR Assay Targeting the SSU rRNA Genes

A quantitative PCR assay using SYBR Green chemistry was previously reported for the detection of *P. falciparum* within the mosquito [37]. This qPCR assay was targeting the multicopy SSU rRNA gene and showed a detection threshold of 10 parasites. The performance of the cox1 qPCR assay was compared to the qPCR assay targeting the SSU rRNA gene developed by Bell and Ranford-Cartwright (2004). Quantitative PCR reactions were processed as described above using our DNA standards as templates and primers from Bell that amplify a 180 bp fragment of the SSU rRNA gene. Standard curves were built as described above.

### Statistical Analysis

Statistical analyses were performed using the R statistical software [51], and significance threshold was set at 0.05. Sensitivity of the qPCR assay was compared to the conventional PCR using Fisher’s exact test. The Cohen’s kappa coefficient κ was calculated to measure the agreement between methods.

A meta-analysis was performed to measure the effect of the M or S molecular form on mosquito infection. Parasite density of the blood donor affects the infection outcomes, giving variation from one feeding to another [52], [53], [54]; thus we used a random-effects model that takes into account the heterogeneity between feedings and balances the assay weights accordingly. In the meta-analysis, an estimate of molecular form effect on the mosquito infection is computed for each gametocyte carrier and a combined estimate of the effect is generated across all assays [55]. For each feeding, estimates of molecular form effect were measured as the odds ratio (OR) and 95% confidence interval (95% CI) for the prevalence of infection, and the standardized mean difference and 95% CI for the infection intensity. The mosquito infection prevalence was defined as the proportion of mosquitoes detected positive by the qPCR assay (number of genomes/µl>1), and the infection intensity as the number of genomes/µl in positive mosquitoes. ORs and mean differences were calculated with the M form mosquitoes as reference; e.g. OR values>1 and positive mean differences indicate higher infection in the M molecular form. The meta-analysis and forest plots were performed using the meta package in R [56]. The difference in prevalence of infection between M and S forms was visualized using a Bland Altman plot, which consists of plotting the difference of the paired proportions of infected mosquitoes in the y-axis against the mean proportion in the x-axis. The difference in prevalence of infection between the two forms was tested using a test of equality of proportions.

## Results

### Mosquito Samples

A total of 990 female mosquitoes successfully fed on *P. falciparum* gametocyte carriers. We genotyped the mosquitoes for the M and S molecular forms, and found 239 (24.1%) M and 748 (75.6%) S and 3 (0.3%) M/S hybrids. The 3 hybrid mosquitoes were from the same locality, they were excluded for the analyses comparing the *P. falciparum* susceptibility of the M and S molecular forms.

### Validation of the cox1 qPCR Assay

**Figure 1:**
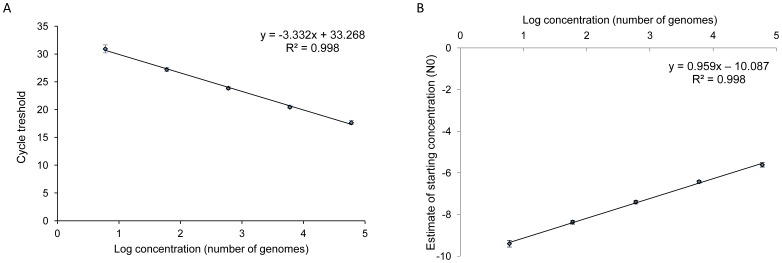
Standard curve of qPCR using serial dilutions of DNAs from cultured parasites. A, Standard curve was obtained by linear regression analysis of Ct values versus log_10_ copy number of cultured parasites in DNA standards (6 to 6×10^4^ genomes/µl). The slope was calculated from 53 independent reactions for each serial dilution. Errors bars show the standard deviation. B, Calibration curve for absolute quantification representing the starting concentrations of DNA standards (N0), expressed as arbitrary fluorescent units, versus log_10_ input copy numbers of cultured parasites (6 to 6×10^4^ genomes/µl). N0 were calculated for each sample of the serial dilutions using the LinRegPCR software. The curve shows a good correlation of the N0 values with the estimate parasite number in serial dilutions (slope = 0.952, and R^2^>0.998). The calibration curve was used to convert the starting concentration of parasites in each mosquito midgut into the number of genomes/µl.

Melting curve analysis of samples showed a single peak, with an average melting temperature (Tm ± sd) at 76.84°C ±0.31. Size and quality of the amplified qPCR products were checked on agarose gel, and showed clear specific bands of 120 bp for both the cultured-parasite and mosquito samples (data not shown). No amplification was obtained with DNAs from mosquito and other malaria species, thus the cox1 qPCR assay allows specific detection of *P. falciparum* which in accordance with previous results [47], [48]. The standard curve generated from a composite of 53 standard curves of 10-fold serial dilutions of parasite-cultured DNA showed the good reproducibility of the qPCR assay (Figure 1A); the standard deviation was <0.75, and the combined standard curve showed a linear relationship with a slope of −3.332 and a regression value (R^2^)>0.998 (Figure 1A). The means of amplification efficiencies per amplicon for cultured parasites and midgut samples were 94.3% (±0.6) and 95.7% (±0.6), respectively, and the difference is not significant (*P* = 0.155). The calibration curve used for absolute quantification of *P. falciparum* parasites within mosquito midguts showed a good correlation of the starting concentrations determined by LinRegPCR with input values from the serial dilution samples (slope = 0.952, and R^2^>0.998; Figure 1B). The limit of detection, as determined on the standard curve of the highest dilution of the parasite-cultured DNA, was 6 genomes/µl, which corresponds to 120 copies/midgut. The cox1 qPCR assay can thus detect as little as one oocyst per midgut, a mature oocyst containing several hundred genomes. The number of *P. falciparum* genomes in qPCR-positive midguts reached up to 137,352 parasites/µl (median: 452, 95% CI: 49–2,513), reflecting a large variation of infection intensity among mosquitoes.

### Comparison of cox1 and SSU rRNA qPCR Assays

**Figure 2:**
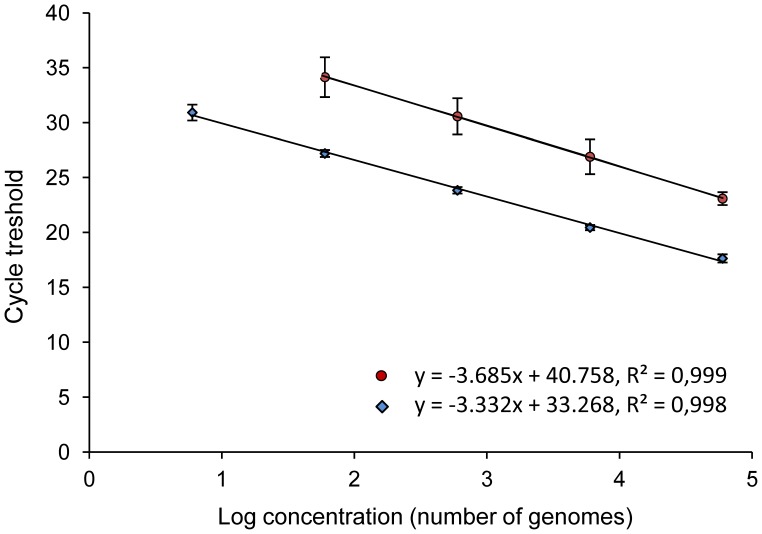
Comparison of standard curves between qPCR assays targeting the cox1 and SSU rRNA genes. Composite plots were obtained by linear regression analysis of Ct values versus log_10_ copy numbers of cultured parasites in DNA standards (6 to 6×10^4^ genomes/µl). The slopes were generated from 53 standard curves for the cox1 assay (blue diamonds) and 38 for the SSU rRNA assay (red circles), respectively. Error bars indicate the standard deviation. The SSU rRNA qPCR assay was not reproducible in reactions containing 6 genomes and was not taken into account in the plot.

We compared the performance of the cox1 qPCR assay with the previously described assay in Bell and Ranford-Cartwright (2004) and composite plots for the two assays are shown in Figure 2. Composite plots were obtained from 19 and 53 standard curves for the SSU rRNA and cox1 qPCR assays, respectively. Bell and Ranford-Cartwright (2004), using SYBR Green chemistry, reported an assay efficiency of 89% and a detection threshold of 10 parasites [37]. Here, using the Pol EvaGreen chemistry for the SSU rRNA qPCR, the endpoint dilution (6 genomes/µl) lacked reproducibility and only 7 replicates (out of 38) for this dilution gave a signal. The cox1 qPCR assay was then more efficient to detect low parasite numbers. Ct values for both assays differed; the *y*-axis intercept points at the smallest dilution were 23.09±0.58 (mean and standard deviation) and 17.63±0.37 for the amplification of the SSU rRNA and cox1 genes, respectively, indicating a higher sensitivity of the cox1 primer set. The qPCR assay efficiencies derived from the slopes of the standard curves (−3.685 and −3.332 for SSU rRNA and cox1 qPCR assays, Figure 2) were 86% and 99%, respectively. The SSU rRNA qPCR assay efficiency was similar to the one described in Bell and Ranford-Cartwright, (89%), but lower to the efficiency obtained with the cox1 qPCR assay.

### Comparison of the cox1 qPCR Assay to the Conventional PCR

Detection of *P. falciparum* in mosquitoes exposed to natural isolates of gametocytes through membrane feedings was performed using a *P. falciparum* specific PCR, while a qPCR assay was developed and used for both detection and quantification of *P. falciparum* parasites in midgut samples. Of the 990 midgut samples, 188 (18.9%) were positive with the classical PCR, 369 (37.3%) with the cox1 qPCR, and the difference is significant (X^2^ = 390.52, 95% CI: 0.74–0.80, *P*<0.001). All samples positive with the conventional PCR yielded a signal with the qPCR. A total of 181 samples negative with the PCR (181/781; 23.2%) were scored positive with the real-time qPCR assay. The measure of the Cohen’s kappa coefficient for the 2 methods is 0.57, indicating moderate agreement between the PCR and the qPCR. The qPCR assay is more sensitive than the conventional PCR, detecting 1.96-fold more *P. falciparum* infected samples.

### Susceptibility of M and S Molecular Forms of *An. gambiae* to *P. falciparum* Infection

**Figure 3:**
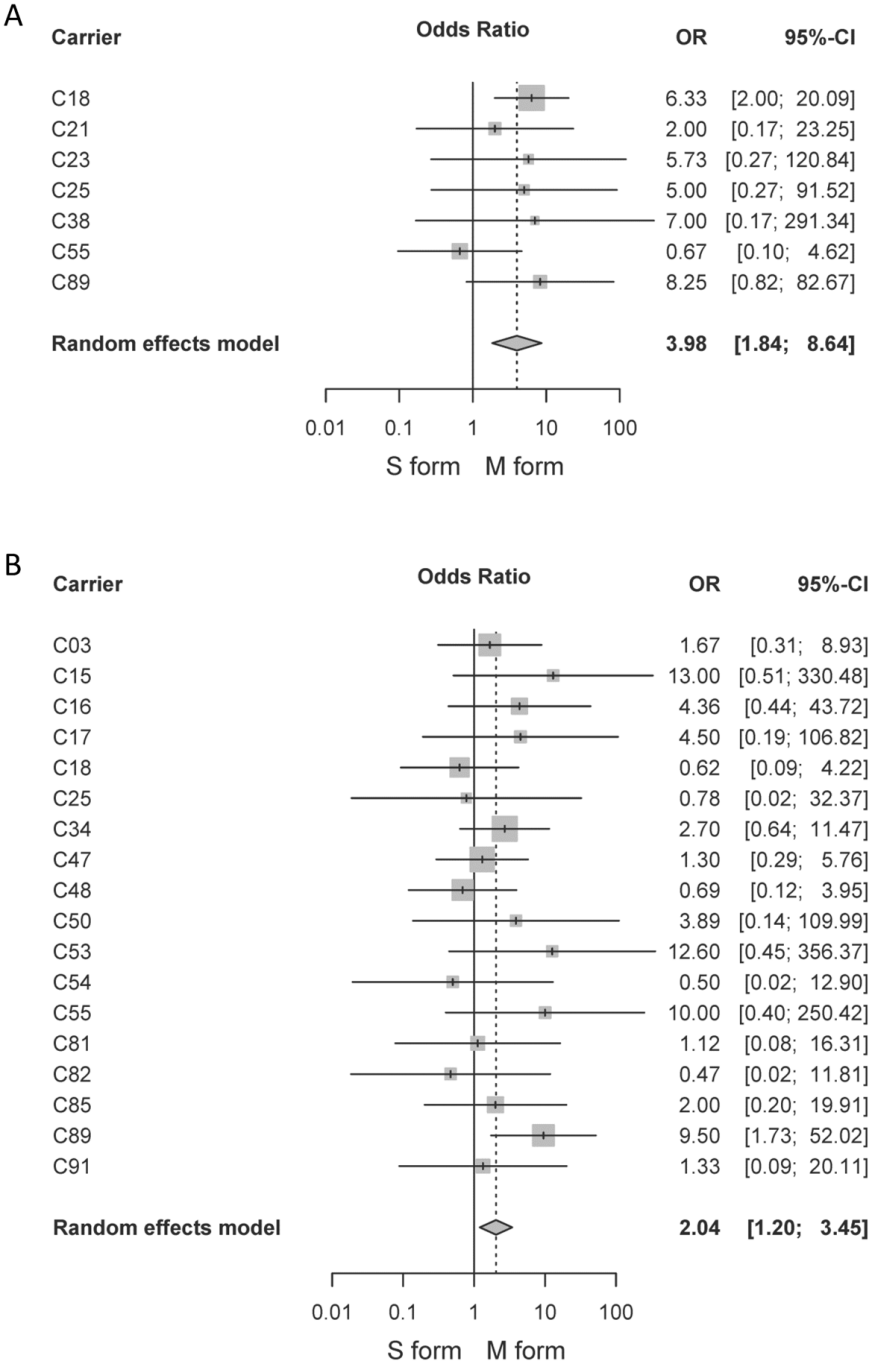
Forest plots for the infection prevalence between the M and S form of *An. gambiae* s.s. in allopatric (A) and sympatric (B) conditions. Each lane corresponds to a feeding on a single gametocyte carrier. Odds ratio (OR) and confidence interval (95% CI) were computed for each carrier and values are shown on the right part of the plot. OR values>1 indicate a higher prevalence of infection in the M form. Grey squares represent the OR estimate for each feeding, and the square size is proportional to the feeding’s weight in the meta-analysis. All feedings were combined in the random effects meta-analysis and the summary OR estimate is indicated at the bottom of the plot by a diamond.

**Figure 4:**
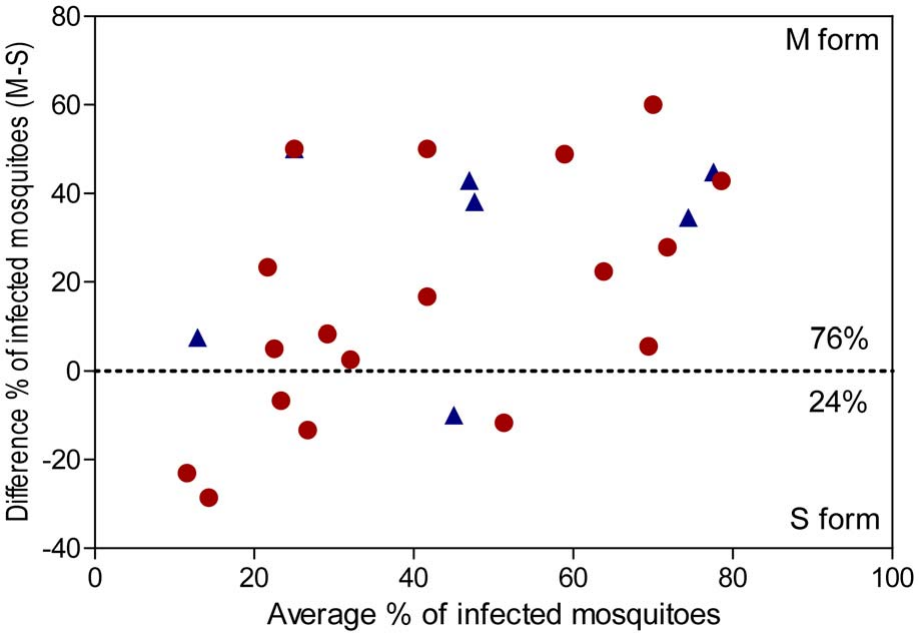
Bland-Altman plot. The plot shows the difference of infection prevalence between the M and S molecular forms in mosquito populations collected in allopatric (blue triangles) and sympatric (red circles) conditions. Each triangle or circle represents an experiment for which M and S mosquitoes were fed on a same blood donor. A positive value indicates a higher infection rate for the M form, and a negative one a higher infection rate for the S form. The M form was more infected than the S one for 76% (19/25) of feedings, and the difference is significant (X^2^ = 11.52, *P*<0.001).

The forest plots for the meta-analysis of the infection prevalence between the M and S forms are shown in Figure 3. The pooled OR estimate using random-effects model was 3.98 (95% CI: 1.84–8.64) and 2.04 (95% CI; 1.20–3.45) for allopatric and sympatric populations, respectively (Figure 3, Table 1). The M molecular form of *An. gambiae* is significantly more susceptible to *P. falciparum* infection than the S form in our studied area, for both allopatric and sympatric populations of mosquitoes (*P* = 0.0005 and *P* = 0.008, respectively, Table 1). We assessed the relative susceptibility of the M and S forms among all feedings (18 feedings for sympatric populations and 7 for allopatric ones) and found that the M form of *An. gambiae* was more infected than the S one for 76% (19/25) (X^2^ = 11.52, *P*<0.001; Figure 4). In allopatric conditions, the M form had higher infection prevalence than the S form for 86% (6/7) of feedings, in sympatric conditions, for 72% (13/18); and the difference is not significant (X^2^ = 0.003, *P* = 0.851). The proportion of infected mosquitoes is higher in the M molecular form for both sympatric and allopatric populations. In contrast, no difference of the infection prevalence was detected between allopatric and sympatric populations of each molecular form (OR = 0.92, 95% CI: 0.36–2.40; and OR = 0.91, 95% CI: 0.38–2.22 for M and S form, respectively; Table 2). The co-occurrence of the other molecular form within the breeding site does not affect the mosquito susceptibility to *P. falciparum* infection. The infection intensity, measured as the number of genomes per µl for each mosquito, was not significant, neither between M and S forms nor between sympatric and allopatric populations (Table 2).

**Table 1:** Results of the meta-analysis on infection prevalence for the different combinations of mosquito populations.

Mosquito comparison	OR	95% CI	Z value	*P* value*
**M allopatric vs. S allopatric**	3.98	1.84; 8.64	3.50	***0.0005***
**M sympatric vs. S sympatric**	2.04	1.20; 3.45	2.65	***0.0080***
**M allopatric vs. M sympatric**	0.92	0.36; 2.40	−0.16	0.8700
**S allopatric vs. S sympatric**	0.91	0.38; 2.22	−0.20	0.8430

* The P value was computed using random effects model. Significant values are indicated in bold italics.

**Table 2:** Results of the meta-analysis on infection intensity for the different combinations of mosquito populations.

Mosquito comparison	Meandiff.	95% CI	Z value	*P* value*
**M allopatric vs. S allopatric**	−133.80	−451.37; 183.77	−0.83	0.4089
**M sympatric vs. S sympatric**	2.65	−115.65; 120.95	0.04	0.9650
**M allopatric vs. M sympatric**	−30.74	−735.93; 674.44	−0.09	0.9319
**S allopatric vs. S sympatric**	84.51	−200.13; 369.15	0.58	0.5606

* The P value was computed using random effects model.

## Discussion

In this study, a real-time qPCR assay targeting the cox1 gene of *P. falciparum* was developed for detection and quantification of *P. falciparum* parasites in the mosquito vector. The assay is reproducible, sensitive and time-saving as compared to other conventional PCR, therefore, providing a promising tool to determine malaria infection parameters in the mosquito. We used the cox1 qPCR assay to investigate the *P. falciparum* infection levels of mosquitoes collected in the field in Cameroon and found different susceptibility between M and S molecular forms of *An. gambiae* s.s.

The real-time PCR is currently widely used for detection and quantification of pathogens in clinical samples for research and diagnostics purposes. The cost of such assays in developing countries may be a limitation for its application to routine diagnosis but many research laboratories are now equipped and the price of reagents is falling; therefore these assays need to be implemented for larger use in monitoring and surveillance efforts. The cox1 qPCR assay presented here offers several advantages over other published assays: 1) the Evagreen dye is a stable non-mutagenic and non-cytotoxic dye, it is compatible with all common real-time PCR cyclers and its cost is two-times less expensive than other commonly used dyes, such as SYBR Green; 2) no fluorescently labeled probe that seriously increases the reaction cost is needed.

The cox1 qPCR assay showed a higher sensitivity than the SSU rRNA qPCR assay previously described in Bell and Ranford-Cartwright [37] and allowed to amplify lower numbers of parasites. The increased sensitivity of the cox1 assay probably results from genome copy number; indeed *P. falciparum* parasites contain numerous mitochondria, ∼20 copies in ring stage parasites, while the SSU rRNA gene has 4–8 copies per individual [39], [40], [41]. Also, our qPCR assay increased the parasite detection in mosquito samples up to 1.96-fold as compared to conventional PCR. The cox1 qPCR assay we developed here may thus have multiple applications for studies aiming at measuring *P. falciparum* transmission. This tool would be useful for the evaluation of the impact of vector control interventions on malaria transmission, as well as for the surveillance of malaria in countries where disease has been declared eradicated recently. Furthermore, clinical trials for the development of TBDs and TBVs assays require accurate measurement of the blocking effect within the mosquito [57]. Our assay with its potential for detecting low parasitaemias, as little as one oocyst per midgut, and its higher throughput design as compared to the current detection methods such as microscopy and conventional PCRs, appears suitable for evaluation assays of TBDs and TBVs on the development of *P. falciparum* within the mosquito in endemic settings.

On the vector side, this qPCR assay will be an important tool to determine the role of different malaria vector species or populations in disease transmission. The current vector control interventions, based on insecticide spraying and protection with impregnated bednets, are targeting the main vector species, such as *An. gambiae* and *An. funestus*; but other species with distinct biological features may be favored by selective pressure and become more efficient vectors in a close future. Out of the 400 described *Anopheles* species, less than 20 are capable of transmitting malaria parasites [58]. Some species are more susceptible to *Plasmodium* infection than others, and even at the population level, their susceptibility differs [21], [22], [59]. The respective role of the different vectors in malaria transmission needs to be clarified, and our assay will be useful to provide accurate estimation of the entomological inoculation rate (EIR) of each vector species or populations; EIR being the key parameter to measure malaria transmission intensity in endemic areas.

We used the cox1 qPCR assay to investigate the *P. falciparum* susceptibility of the M and S molecular forms of *An. gambiae* s.s. in Cameroon. Mosquitoes were collected at immature stages in natural breeding sites and reared in the water of their aquatic habitat until the emergence to maintain natural conditions. Indeed, the midgut microbiota of the mosquito plays an important role in modulating malaria infection, and we have previously shown that the bacterial flora of laboratory-reared mosquitoes is particularly poor [60], [61]. Field-derived female mosquitoes were experimentally infected on blood from naturally-infected gametocyte carriers and dissected 8 days upon feeding. Their midgut was recovered for parasite detection and quantification using the qPCR assay, and for molecular form identification using a diagnostic-PCR protocol.

We found 3 M/S hybrids among the mosquitoes collected at immature stages in natural breeding sites, and this is the first report of hybrids in Cameroon. M/S hybrids are rarely observed in natural settings but several recent studies reported levels of hybridization higher than expected for cryptic species [19], [20], [22], [59], [62], [63], [64] ). Subgroups of *An. gambiae* s.s. are likely occurring in nature, that are missed with standard sampling methods, and characterizing these subgroups will be a challenge as they may have an importance in malaria transmission in particular areas [59].

Our results reveal a higher prevalence of infection in the M molecular form of *An. gambiae* s.s. in Cameroon. This finding contrasts with previous reports, where in Senegal, higher infection rates were found in the S molecular form, and in Mali similar levels of infection were reported for the M and S forms [21], [22]. In addition, in Senegal, the authors reported higher oocyst loads in the S molecular form but we did not find any difference in the infection intensities of the M and S forms in Cameroon. Our study and the previous ones used different protocols: in Senegal, laboratory-reared progenies were experimentally infected through membrane and oocysts counted by microscopy; in Mali, field-collected females of unknown parasite exposure were analyzed by CSP-ELISA. These differences may account for a certain level of variability but also other factors. Indeed, we infected in this study mosquitoes collected in the field while in Senegal mosquitoes were reared for 2–3 generations in insectary before the infectious feeding. We have previously shown the importance of the natural midgut microbiota on *P. falciparum* infection [60]. The mosquito microbiota is particularly poor in insectary conditions and infections of laboratory-reared mosquitoes probably not reflect the mosquito susceptibility *in natura*. In Mali, parasite infections were recorded in salivary glands of resting mosquitoes collected inside houses which render the comparison between studies difficult. The age structure of mosquito specimens was unknown. In addition, sporozoites are massively killed in the mosquito hemolymph, possibly leading to differences between oocyst prevalence and sporozoitic index [65]. Nonetheless, these results showed distinct patterns in different ecological settings, and highlight the importance of determining infection parameters at local scales.

The mosquito susceptibility to malaria infection relies on both genetic and environmental factors [26], [43], [60], [66], [67], [68]. In this study, we compared the prevalence of infection between M and S forms for sympatric and allopatric populations of mosquitoes and found a higher proportion of infected mosquitoes in the M form for both conditions. The distribution of *An. gambiae* is markedly associated with human activities but the M and S forms of *An. gambiae* s.s. have distinct ecological preferences and more often colonize distinct habitats [19], [20], [69]. In Cameroon, the M molecular form is more adapted to urbanized, polluted environments while the S form is predominant in rural and semi-urban areas, both forms occurring in sympatry in overlapping belts [20], [70], [71]. The difference in the mosquito susceptibility between the molecular forms could result from this geographical partitioning, some biotic factors putatively conferring refractoriness in the different larval habitats [60]. Accordingly, the difference in the prevalence of infection is larger for allopatric than for sympatric populations. However, no difference intra-form was found between mosquitoes collected in allopatry and sympatry, and this indicates that the composition of the breeding sites is not the main factor modulating the mosquito susceptibility. Widespread genomic divergence between M and S forms was detected upon whole genome comparison [15], this may explain, in part, the differential infection levels.

In Mali, different infection rates were recorded among chromosomal forms of mosquitoes [22]. In our study, *An. gambiae* mosquitoes are of Forest form, with standard 2La inversion, and the susceptibility to malaria infection is then not influenced by the mosquito karyotype. Genetic diversity of *An. gambiae* is higher in immune genes [72], and polymorphisms at immune-related genes have been linked to the mosquito susceptibility [43], [66], [73]. Particularly, the complement-like thioester-containing protein 1 (TEP1), an anti-parasitic factor controlling infection in rodent and human malaria, exhibits extremely large nucleotide diversity and analysis of the sequence polymorphisms has lead to the distinction of different alleles, 2 resistant (R1, R2) and 2 susceptible (S1, S2) [66], [68]. In our studied area, preliminary studies indicate that the TEP1 S1 allele is predominant and the R1 resistant allele is only rarely found; however, the S2 allele is more frequent in the M form and the R2 in the S one (Levashina and Morlais, unpublished results). Further studies to correlate the mosquito infection with the mosquito genotypes at TEP1 loci are under process and the results would help to better understand the effect of the genetic polymorphism on the difference of susceptibility to malaria infection between the M and S forms in Cameroon.

Finally, we have developed a cox1 qPCR assay that allows sensitive detection and quantification of *P. falciparum* in biological samples. This qPCR assay provides a useful tool for monitoring of the *P. falciparum* burden, and for measuring the impact of vector control interventions in malaria endemic countries. Using this assay, we showed that, in Cameroon, the M molecular form of *An. gambiae* s.s. has a greater susceptibility to malaria infection. Our results suggest that the mosquito susceptibility is controlled by genome*environment interactions and point out the importance of characterizing mosquito populations in malaria endemic areas. Indeed, the distribution of the molecular forms of *An. gambiae* s.s. may impact on the malaria epidemiology, and in Cameroon, by increasing malaria risk in urban settings where the M form is more prevalent. Urban malaria in Africa where city populations are growing is an important public health problem and understanding the factors that influence the mosquito infection would help to better target control interventions.

## References

[1] WHO World Health Organization. World malaria report 2011 (2011) Available: http://www.who.int/malaria/world_malaria_report_2011.

[2] Goals MD. Available: wwwunorg/millenniumgoals/.

[3] Alonso PL, Brown G, Arevalo-Herrera M, Binka F, Chitnis C, et al (2011) A research agenda to underpin malaria eradication. PLoS Med 8: e1000406. 21311579 10.1371/journal.pmed.1000406 PMC3026687

[4] Dondorp AM, Yeung S, White L, Nguon C, Day NP, et al (2010) Artemisinin resistance: current status and scenarios for containment. Nat Rev Microbiol 8: 272–280. 20208550 10.1038/nrmicro2331

[5] Kudom AA, Mensah BA, Agyemang TK (2012) Characterization of mosquito larval habitats and assessment of insecticide-resistance status of Anopheles gambiae senso lato in urban areas in southwestern Ghana. J Vector Ecol 37: 77–82. 22548539 10.1111/j.1948-7134.2012.00202.x

[6] Ranson H, N’Guessan R, Lines J, Moiroux N, Nkuni Z, et al (2011) Pyrethroid resistance in African anopheline mosquitoes: what are the implications for malaria control? Trends Parasitol 27: 91–98. 20843745 10.1016/j.pt.2010.08.004

[7] Coetzee M, Craig M, le Sueur D (2000) Distribution of African malaria mosquitoes belonging to the Anopheles gambiae complex. Parasitol Today 16: 74–77. 10652493 10.1016/s0169-4758(99)01563-x

[8] Onyabe DY, Conn JE (2001) The distribution of two major malaria vectors, Anopheles gambiae and Anopheles arabiensis, in Nigeria. Mem Inst Oswaldo Cruz 96: 1081–1084. 11784926 10.1590/s0074-02762001000800009

[9] White GB (1974) Anopheles gambiae complex and disease transmission in Africa. Trans R Soc Trop Med Hyg 68: 278–301. 4420769 10.1016/0035-9203(74)90035-2

[10] Favia G, della Torre A, Bagayoko M, Lanfrancotti A, Sagnon N, et al (1997) Molecular identification of sympatric chromosomal forms of Anopheles gambiae and further evidence of their reproductive isolation. Insect Mol Biol 6: 377–383. 9359579 10.1046/j.1365-2583.1997.00189.x

[11] Gentile G, Slotman M, Ketmaier V, Powell JR, Caccone A (2001) Attempts to molecularly distinguish cryptic taxa in Anopheles gambiae s.s. Insect Mol Biol 10: 25–32. 11240634 10.1046/j.1365-2583.2001.00237.x

[12] Slotman MA, Della Torre A, Calzetta M, Powell JR (2005) Differential introgression of chromsomal regions between Anopheles gambiae and An. arabiensis. Am J Trop Med Hyg 73: 326–335. 16103599

[13] Stump AD, Shoener JA, Costantini C, Sagnon N, Besansky NJ (2005) Sex-linked differentiation between incipient species of Anopheles gambiae. Genetics 169: 1509–1519. 15654109 10.1534/genetics.104.035303 PMC1449544

[14] Turner TL, Hahn MW, Nuzhdin SV (2005) Genomic islands of speciation in Anopheles gambiae. PLoS Biol 3: e285. 16076241 10.1371/journal.pbio.0030285 PMC1182689

[15] Lawniczak MK, Emrich SJ, Holloway AK, Regier AP, Olson M, et al (2010) Widespread divergence between incipient Anopheles gambiae species revealed by whole genome sequences. Science 330: 512–514. 20966253 10.1126/science.1195755 PMC3674514

[16] della Torre A, Fanello C, Akogbeto M, Dossou-yovo J, Favia G, et al (2001) Molecular evidence of incipient speciation within Anopheles gambiae s.s. in West Africa. Insect Mol Biol 10: 9–18. 11240632 10.1046/j.1365-2583.2001.00235.x

[17] Wondji C, Simard F, Fontenille D (2002) Evidence for genetic differentiation between the molecular forms M and S within the Forest chromosomal form of Anopheles gambiae in an area of sympatry. Insect Mol Biol 11: 11–19. 11841498 10.1046/j.0962-1075.2001.00306.x

[18] Lawniczak MKN, Emrich SJ, Holloway AK, Regier AP, Olson M, et al (2010) Widespread Divergence Between Incipient Anopheles gambiae Species Revealed by Whole Genome Sequences. Science 330: 512–514. 20966253 10.1126/science.1195755 PMC3674514

[19] Costantini C, Ayala D, Guelbeogo WM, Pombi M, Some CY, et al (2009) Living at the edge: biogeographic patterns of habitat segregation conform to speciation by niche expansion in Anopheles gambiae. BMC Ecol 9: 16. 19460144 10.1186/1472-6785-9-16 PMC2702294

[20] Simard F, Ayala D, Kamdem GC, Pombi M, Etouna J, et al (2009) Ecological niche partitioning between Anopheles gambiae molecular forms in Cameroon: the ecological side of speciation. BMC Ecol 9: 17. 19460146 10.1186/1472-6785-9-17 PMC2698860

[21] Ndiath MO, Cohuet A, Gaye A, Konate L, Mazenot C, et al (2011) Comparative susceptibility to Plasmodium falciparum of the molecular forms M and S of Anopheles gambiae and Anopheles arabiensis. Malar J 10: 269. 21929746 10.1186/1475-2875-10-269 PMC3184635

[22] Trout Fryxell RT, Nieman CC, Fofana A, Lee Y, Traore SF, et al (2012) Differential Plasmodium falciparum infection of Anopheles gambiae s.s. molecular and chromosomal forms in Mali. Malar J 11: 133. 22540973 10.1186/1475-2875-11-133 PMC3441388

[23] Sinden RE (1999) Plasmodium differentiation in the mosquito. Parassitologia 41: 139–148. 10697846

[24] Levashina EA (2004) Immune responses in Anopheles gambiae. Insect Biochem Mol Biol 34: 673–678. 15242708 10.1016/j.ibmb.2004.03.020

[25] Michel K, Kafatos FC (2005) Mosquito immunity against Plasmodium. Insect Biochem Mol Biol 35: 677–689. 15894185 10.1016/j.ibmb.2005.02.009

[26] Mitri C, Vernick KD (2012) Anopheles gambiae pathogen susceptibility: the intersection of genetics, immunity and ecology. Curr Opin Microbiol. 10.1016/j.mib.2012.04.001 PMC3404259 22538050

[27] Babiker HA, Abdel-Wahab A, Ahmed S, Suleiman S, Ranford-Cartwright L, et al (1999) Detection of low level Plasmodium falciparum gametocytes using reverse transcriptase polymerase chain reaction. Mol Biochem Parasitol 99: 143–148. 10215031 10.1016/s0166-6851(98)00175-3

[28] Berry A, Benoit-Vical F, Fabre R, Cassaing S, Magnaval JF (2008) PCR-based methods to the diagnosis of imported malaria. Parasite 15: 484–488. 18814727 10.1051/parasite/2008153484

[29] Mangold KA, Manson RU, Koay ES, Stephens L, Regner M, et al (2005) Real-time PCR for detection and identification of Plasmodium spp. J Clin Microbiol 43: 2435–2440. 15872277 10.1128/JCM.43.5.2435-2440.2005 PMC1153761

[30] Rougemont M, Van Saanen M, Sahli R, Hinrikson HP, Bille J, et al (2004) Detection of four Plasmodium species in blood from humans by 18S rRNA gene subunit-based and species-specific real-time PCR assays. J Clin Microbiol 42: 5636–5643. 15583293 10.1128/JCM.42.12.5636-5643.2004 PMC535226

[31] Schoone GJ, Oskam L, Kroon NC, Schallig HD, Omar SA (2000) Detection and quantification of Plasmodium falciparum in blood samples using quantitative nucleic acid sequence-based amplification. J Clin Microbiol 38: 4072–4075. 11060070 10.1128/jcm.38.11.4072-4075.2000 PMC87543

[32] Snounou G, Viriyakosol S, Jarra W, Thaithong S, Brown KN (1993) Identification of the four human malaria parasite species in field samples by the polymerase chain reaction and detection of a high prevalence of mixed infections. Mol Biochem Parasitol 58: 283–292. 8479452 10.1016/0166-6851(93)90050-8

[33] Steenkeste N, Incardona S, Chy S, Duval L, Ekala MT, et al (2009) Towards high-throughput molecular detection of Plasmodium: new approaches and molecular markers. Malar J 8: 86. 19402894 10.1186/1475-2875-8-86 PMC2686730

[34] Schneider P, Wolters L, Schoone G, Schallig H, Sillekens P, et al (2005) Real-time nucleic acid sequence-based amplification is more convenient than real-time PCR for quantification of Plasmodium falciparum. J Clin Microbiol 43: 402–405. 15635001 10.1128/JCM.43.1.402-405.2005 PMC540116

[35] Arez AP, Lopes D, Pinto J, Franco AS, Snounou G, et al (2000) Plasmodium sp.: optimal protocols for PCR detection of low parasite numbers from mosquito (Anopheles sp.) samples. Exp Parasitol 94: 269–272. 10831396 10.1006/expr.2000.4496

[36] Bass C, Nikou D, Blagborough AM, Vontas J, Sinden RE, et al (2008) PCR-based detection of Plasmodium in Anopheles mosquitoes: a comparison of a new high-throughput assay with existing methods. Malar J 7: 177. 18793416 10.1186/1475-2875-7-177 PMC2553798

[37] Bell AS, Ranford-Cartwright LC (2004) A real-time PCR assay for quantifying Plasmodium falciparum infections in the mosquito vector. Int J Parasitol 34: 795–802. 15157762 10.1016/j.ijpara.2004.03.008

[38] Rao RU, Huang Y, Bockarie MJ, Susapu M, Laney SJ, et al (2009) A qPCR-based multiplex assay for the detection of Wuchereria bancrofti, Plasmodium falciparum and Plasmodium vivax DNA. Trans R Soc Trop Med Hyg 103: 365–370. 18801545 10.1016/j.trstmh.2008.07.012 PMC2713189

[39] McCutchan TF, Li J, McConkey GA, Rogers MJ, Waters AP (1995) The cytoplasmic ribosomal RNAs of Plasmodium spp. Parasitol Today 11: 134–138. 15275356 10.1016/0169-4758(95)80132-4

[40] Krungkrai J (2004) The multiple roles of the mitochondrion of the malarial parasite. Parasitology 129: 511–524. 15552397 10.1017/s0031182004005888

[41] Preiser PR, Wilson RJ, Moore PW, McCready S, Hajibagheri MA, et al (1996) Recombination associated with replication of malarial mitochondrial DNA. EMBO J 15: 684–693. 8599952 PMC449987

[42] Service MN (1977) A critical review of procedure for sampling populations of adult mosquitoes. Bulletin of Entomological Research 67: 343–382.

[43] Harris C, Lambrechts L, Rousset F, Abate L, Nsango SE, et al (2010) Polymorphisms in Anopheles gambiae Immune Genes Associated with Natural Resistance to Plasmodium falciparum. PLoS Pathog 6: e1001112. 20862317 10.1371/journal.ppat.1001112 PMC2940751

[44] Mendes AM, Schlegelmilch T, Cohuet A, Awono-Ambene P, De Iorio M, et al (2008) Conserved mosquito/parasite interactions affect development of Plasmodium falciparum in Africa. PLoS Pathog 4: e1000069. 18483558 10.1371/journal.ppat.1000069 PMC2373770

[45] Fabre R, Berry A, Morassin B, Magnaval JF (2004) Comparative assessment of conventional PCR with multiplex real-time PCR using SYBR Green I detection for the molecular diagnosis of imported malaria. Parasitology 128: 15–21. 15002899 10.1017/s0031182003004219

[46] Fanello C, Santolamazza F, della Torre A (2002) Simultaneous identification of species and molecular forms of the Anopheles gambiae complex by PCR-RFLP. Med Vet Entomol 16: 461–464. 12510902 10.1046/j.1365-2915.2002.00393.x

[47] Bourgeois N, Boutet A, Bousquet PJ, Basset D, Douard-Enault C, et al (2010) Comparison of three real-time PCR methods with blood smears and rapid diagnostic test in Plasmodium sp. infection. Clin Microbiol Infect 16: 1305–1311. 19840032 10.1111/j.1469-0691.2009.02933.x

[48] Elsayed S, Plewes K, Church D, Chow B, Zhang K (2006) Use of molecular beacon probes for real-time PCR detection of Plasmodium falciparum and other plasmodium species in peripheral blood specimens. J Clin Microbiol 44: 622–624. 16455928 10.1128/JCM.44.2.622-624.2006 PMC1392706

[49] Ramakers C, Ruijter JM, Deprez RH, Moorman AF (2003) Assumption-free analysis of quantitative real-time polymerase chain reaction (PCR) data. Neurosci Lett 339: 62–66. 12618301 10.1016/s0304-3940(02)01423-4

[50] Ruijter JM, Ramakers C, Hoogaars WM, Karlen Y, Bakker O, et al (2009) Amplification efficiency: linking baseline and bias in the analysis of quantitative PCR data. Nucleic Acids Res 37: e45. 19237396 10.1093/nar/gkp045 PMC2665230

[51] Team RC (2012) R: A Language and Environment for Statistical Computing. Vienna, Austria: R Foundation for Statistical Computing.

[52] Boudin C, Van Der Kolk M, Tchuinkam T, Gouagna C, Bonnet S, et al (2004) Plasmodium falciparum transmission blocking immunity under conditions of low and high endemicity in Cameroon. Parasite Immunol 26: 105–110. 15225297 10.1111/j.0141-9838.2004.00689.x

[53] Schneider P, Bousema T, Omar S, Gouagna L, Sawa P, et al (2006) (Sub)microscopic Plasmodium falciparum gametocytaemia in Kenyan children after treatment with sulphadoxine-pyrimethamine monotherapy or in combination with artesunate. Int J Parasitol 36: 403–408. 16500657 10.1016/j.ijpara.2006.01.002

[54] Graves PM (1980) Studies on the use of a membrane feeding technique for infecting Anopheles gambiae with Plasmodium falciparum. Trans R Soc Trop Med Hyg 74: 738–742. 7010696 10.1016/0035-9203(80)90189-3

[55] Borenstein M, Hedges LV, Higgins JPT, Rothstein HR, editors (2009) Introduction to meta-analysis: Wiley.

[56] Schwarzer G (2012) meta: Meta-Analysis with R. Available: http://CRAN.R-project.org/package=meta.

[57] Sinden RE, Carter R, Drakeley C, Leroy D (2012) The biology of sexual development of Plasmodium: the design and implementation of transmission-blocking strategies. Malaria Journal 11. 10.1186/1475-2875-11-70 PMC3315749 22424474

[58] Harbach RE (2004) The classification of genus Anopheles (Diptera: Culicidae): a working hypothesis of phylogenetic relationships. Bull Entomol Res 94: 537–553. 15541193 10.1079/ber2004321

[59] Riehle MM, Guelbeogo WM, Gneme A, Eiglmeier K, Holm I, et al (2011) A cryptic subgroup of Anopheles gambiae is highly susceptible to human malaria parasites. Science 331: 596–598. 21292978 10.1126/science.1196759 PMC3065189

[60] Boissiere A, Tchioffo MT, Bachar D, Abate L, Marie A, et al (2012) Midgut Microbiota of the Malaria Mosquito Vector Anopheles gambiae and Interactions with Plasmodium falciparum Infection. PLoS Pathog 8: e1002742. 22693451 10.1371/journal.ppat.1002742 PMC3364955

[61] Cirimotich CM, Dong Y, Clayton AM, Sandiford SL, Souza-Neto JA, et al (2011) Natural microbe-mediated refractoriness to Plasmodium infection in Anopheles gambiae. Science 332: 855–858. 21566196 10.1126/science.1201618 PMC4154605

[62] Wondji C, Frederic S, Petrarca V, Etang J, Santolamazza F, et al (2005) Species and populations of the Anopheles gambiae complex in Cameroon with special emphasis on chromosomal and molecular forms of Anopheles gambiae s.s. J Med Entomol 42: 998–1005. 16465741 10.1093/jmedent/42.6.998

[63] Caputo B, Santolamazza F, Vicente JL, Nwakanma DC, Jawara M, et al (2011) The “far-west” of Anopheles gambiae molecular forms. PLoS One 6: e16415. 21347223 10.1371/journal.pone.0016415 PMC3039643

[64] Oliveira E, Salgueiro P, Palsson K, Vicente JL, Arez AP, et al (2008) High levels of hybridization between molecular forms of Anopheles gambiae from Guinea Bissau. J Med Entomol 45: 1057–1063. 19058629 10.1603/0022-2585(2008)45[1057:hlohbm]2.0.co;2

[65] Hillyer JF, Barreau C, Vernick KD (2007) Efficiency of salivary gland invasion by malaria sporozoites is controlled by rapid sporozoite destruction in the mosquito haemocoel. Int J Parasitol 37: 673–681. 17275826 10.1016/j.ijpara.2006.12.00 PMC1905829

[66] Blandin SA, Wang-Sattler R, Lamacchia M, Gagneur J, Lycett G, et al (2009) Dissecting the genetic basis of resistance to malaria parasites in Anopheles gambiae. Science 326: 147–150. 19797663 10.1126/science.1175241 PMC2959166

[67] Collins FH, Sakai RK, Vernick KD, Paskewitz S, Seeley DC, et al (1986) Genetic selection of a Plasmodium-refractory strain of the malaria vector Anopheles gambiae. Science 234: 607–610. 3532325 10.1126/science.3532325

[68] White BJ, Lawniczak MK, Cheng C, Coulibaly MB, Wilson MD, et al (2011) Adaptive divergence between incipient species of Anopheles gambiae increases resistance to Plasmodium. Proc Natl Acad Sci U S A 108: 244–249. 21173248 10.1073/pnas.1013648108 PMC3017163

[69] Gimonneau G, Pombi M, Choisy M, Morand S, Dabire RK, et al (2011) Larval habitat segregation between the molecular forms of the mosquito Anopheles gambiae in a rice field area of Burkina Faso, West Africa. Med Vet Entomol 26: 9–17. 21501199 10.1111/j.1365-2915.2011.00957.x PMC3140611

[70] Antonio-Nkondjio C, Fossog BT, Ndo C, Djantio BM, Togouet SZ, et al (2011) Anopheles gambiae distribution and insecticide resistance in the cities of Douala and Yaounde (Cameroon): influence of urban agriculture and pollution. Malar J 10: 154. 21651761 10.1186/1475-2875-10-154 PMC3118161

[71] Kamdem C, Tene Fossog B, Simard F, Etouna J, Ndo C, et al (2012) Anthropogenic Habitat Disturbance and Ecological Divergence between Incipient Species of the Malaria Mosquito Anopheles gambiae. PLoS One 9: e39453. 10.1371/journal.pone.0039453 PMC3382172 22745756

[72] Morlais I, Poncon N, Simard F, Cohuet A, Fontenille D (2004) Intraspecific nucleotide variation in Anopheles gambiae: new insights into the biology of malaria vectors. Am J Trop Med Hyg 71: 795–802. 15642974

[73] Mitri C, Jacques JC, Thiery I, Riehle MM, Xu J, et al (2009) Fine pathogen discrimination within the APL1 gene family protects Anopheles gambiae against human and rodent malaria species. PLoS Pathog 5: e1000576. 19750215 10.1371/journal.ppat.1000576 PMC2734057

