# Correction: Application of a qPCR Assay in the Investigation of Susceptibility to Malaria Infection of the M and S Molecular Forms of *An*. *gambiae* s.s. in Cameroon

**DOI:** 10.1371/journal.pone.0196956

**Published:** 2018-05-01

**Authors:** Anne Boissière, Geoffrey Gimonneau, Majoline T. Tchioffo, Luc Abate, Albert Bayibeki, Parfait H. Awono-Ambéné, Sandrine E. Nsango, Isabelle Morlais

The following information is missing from the Funding section: This study was supported by the ERC Starting Grant Malares (Grant No. 260918).
